# Risk factors for in-hospital mortality after emergency colorectal surgery in octogenarians: results of a cohort study from a referral center

**DOI:** 10.1007/s00384-023-04565-9

**Published:** 2023-11-21

**Authors:** Guillaume Mathis, Alfonso Lapergola, Florent Alexandre, Guillaume Philouze, Didier Mutter, Antonio D’Urso

**Affiliations:** 1 Visceral and Digestive Surgery Department, University Hospital, Strasbourg, France; 2 IRCAD (Research Institute against Digestive Cancer), Strasbourg, France; 3 IHU (Institut Hospitalo-Universitaire/University Hospital Institute), Strasbourg, France; 4 Department of Surgery, Sapienza University Hospital, Rome, Italy

**Keywords:** Colorectal, Surgery, Emergency, Octogenarians

## Abstract

**Purpose:**

The objective of this study was to investigate predictive factors of mortality in emergency colorectal surgery in octogenarian patients.

**Methods:**

It is a retrospective cohort study conducted at a single-institution tertiary referral center. Consecutive patients who underwent emergency colorectal surgery between January 2015 and January 2020 were identified. The primary endpoint was 30-day mortality. Univariate and multivariate analyses were performed using a logistic regression model.

**Results:**

A total of 111 patients were identified (43 men, 68 women). Mean age was 85.7 ± 3.7 years (80–96). Main diagnoses included complicated sigmoiditis in 38 patients (34.3%), cancer in 35 patients (31.5%), and ischemic colitis in 31 patients (27.9%). An ASA score of 3 or higher was observed in 88.3% of patients. The mean Charlson score was 5.9. The Possum score was 35.9% for mortality and 79.3% for morbidity. The 30-day mortality rate was 25.2%. Univariate analysis of preoperative risk factors for mortality shows that the history of valvular heart disease (p = 0.008), intensive care unit provenance (p = 0.003), preoperative sepsis (p < 0.001), diagnosis of ischemic colitis (p = 0.012), creatinine (p = 0.006) and lactate levels (p = 0.01) were significantly associated with 30-day mortality, and patients coming from home had a lower 30-day mortality rate (p = 0.018). Intraoperative variables associated with 30-day mortality included ileostomy creation (p = 0.022) and temporary laparostomy (p = 0.004). At multivariate analysis, only lactate (p = 0.032) and creatinine levels (p = 0.027) were found to be independent predictors of 30-day mortality, home provenance was an independent protective factor (p = 0.004). Mean follow-up was 3.4 years. Survival at 1 and 3 years was 57.6 and 47.7%.

**Conclusion:**

Emergency colorectal surgery is challenging. However, age should not be a contraindication. The 30-day mortality rate (25.2%) is one of the lowest in the literature. Hyperlactatemia (> 2mmol/L) and creatinine levels appear to be independent predictors of mortality.

## Introduction

Life expectancy in the general population, in developed countries, has increased by almost 30 years over the past two centuries [1].

The segment of the population over the age of 80, referred to as ‘octogenarians’, is the one that has increased most rapidly. Today, it represents a major public health concern.

The increase in life expectancy has led to the development of the so-called “geriatric” surgery, which is progressively becoming one of the largest portions of the activity in everyday surgical practice. In some colorectal surgery studies, octogenarians reach 25% of the population [2]. Consequently, the medical community’s interest in this group of patients has progressively increased in order to improve their overall management.

Advanced age induces many changes and can alter physiological response to different procedures [3], hence putting elderly patients undergoing surgery at a greater risk of suffering major postoperative complications [4].

Various scores exist to assess the patient’s health status prior to surgery and to stratify the risk of postoperative morbidity and mortality (ASA score, Charlson score, POSSUM score, etc.). However, these scores were not specifically developed for a geriatric population.

The management of the surgical patient has improved significantly over the years. Postoperative complications and mortality after elective colorectal surgery in the general and elderly population have improved tremendously over the last 20 years passing from historical rates of 3.4 to 1.7% [5], also made possible with the introduction and development of ERAS (Enhanced Recovery After Surgery) protocols as recently confirmed by the POWER study [6]. In contrast, in case of emergency operations, especially in patients aged over 80 years, surgery is still burdened by very high mortality rates ranging from 20 to 44% [7].

Improving outcomes of elderly patients in emergency surgery is particularly challenging, and improvements in patient evaluation in emergency situations are crucial to ensure the best outcomes.

The objective of our study is to investigate predictive factors of 30-day mortality in the setting of emergency colorectal surgery in patients aged 80 years and older.

## Materials and methods

A cohort of consecutive patients aged 80 years or more who underwent emergency colorectal surgery between January 2015 and January 2020 at the Visceral and Digestive Surgery Department of the University Hospital of Strasbourg (Nouvel Hôpital Civil) were screened for a retrospective analysis.

Inclusion criteria were emergency colorectal surgery intervention and age ≥80 years.

Exclusion criteria were non-colorectal intervention, discharge colostomy without resection, urgent reoperation complicating scheduled surgery.

Prospectively collected clinicodemographic data (i.e., sex, age, patient’s origin, body mass index, ASA score, Charlson score, POSSUM score, heart rate, blood pressure, saturation, temperature), preoperative laboratory values (i.e., CRP, white blood cell count, hemoglobin, platelet count, creatinine, bilirubin, lactate), intraoperative data (i.e., wound class, procedure type, associated procedures, restoration of bowel continuity, placement of a stoma, need for a temporary laparostomy by means of VAC®, operating time) were analyzed retrospectively.

The primary outcome was thirty-day postoperative mortality. Secondary outcomes were postoperative morbidity and survival rate at 1 and 3 years.

According to the Clavien-Dindo classification [8], a minor complication was considered in case of grades I and II whereas a major complication was considered in case of grade > III.

Continuous variables were reported as mean ± standard deviation (SD) and categorical variables in numbers and percentage points, unless otherwise specified. Continuous variables were tested for normal distribution (Kolmogorov-Smirnov test). In the two-group comparison, non-parametric continuous variables were compared with the Mann-Whitney U test whereas a Student’s t-test was used for parametric variables. When more than two groups were compared, a Kruskal-Wallis rank sum test was used for non-parametric continuous variables whereas an ANOVA test was chosen for parametric variables. Paired comparison of qualitative variables was performed with a chi-square test or Fischer’s test. Univariate analyses were performed to determine the association between variables and postoperative mortality. All variables with p < 0.10 at univariate analysis were included in a stepwise multivariate logistic regression model to examine the predictive ability for postoperative mortality. All reported p values were two-tailed, and a p value < 0.05 was required to conclude statistical significance.

Statistical analyses were performed using IBM SPSS v. 25.

## Results

### Clinicodemographic and intraoperative data

From January 2015 to January 2020, a total of 111 consecutive patients underwent an emergency colorectal surgery procedure and met inclusion criteria for the analysis.

The mean age of the population was 85.7 ± 3.7 years (80–96). There were 38.7% of men (n = 43) and 61.3% of women (n = 68).

Arterial hypertension (n = 68, 61.3%), ischemic heart disease (n = 26, 23.4%), and diabetes (n = 22, 19.8%) were the most frequently presented comorbidities.

An ASA score of 3 or higher was observed in 88.3% of the population. The POSSUM score analysis showed a 35.9% probability for mortality and 79.3% for morbidity.

One third of the population (33.3%) presented a Charlson score of 5 (n = 37), followed by 25.2% of the population presenting a score of 6 (n = 28). Additionally, 25 patients had a score of 4 (22.5%), 10 patients a score of 7 (9.1%), 7 patients a score of 8 (6.3%), 2 patients a score of 10 (1.8%), while 1 patient presented a score of 9 (0.9%) and 1 patient a score of 11 (0.9%).

More than half of the patients came from home (n = 61, 54.9%), 24 patients were transferred from another department or an outpatient center (21.7%) while the rest of patients were already hospitalized in an intensive care unit (n = 14, 12.6%) or came from a nursing home or a long-term care and skilled nursing facility (n = 12, 10.8%). Detailed demographic and comorbidity data are reported in Table 1.

**Table 1:** Clinico-demographic data

Variable	Overall population (n = 111)	Survivor (n = 83)	Non-Survivors (n = 28)	*p*
Age	85.7 ± 3.7	85.4 ± 3.5	85.6 ± 4.1	*0.840*
Men, N (%)	43 (38.7)	32 (38.6)	11 (39.3)	*0.945*
Women, N (%)	68 (61.3)	51 (61.4)	17 (60.7)	*0.945*
BMI	24.8 ± 4.6	24.8 ± 4.4	24.8 ± 5.2	*0.980*
ASA Score				
ASA 1, N (%)	0 (0.0)	0 (0.0)	0 (0.0)	
ASA 2, N (%)	13 (11.7)	11 (13.2)	2 (7.1)	*0.385*
ASA 3, N (%)	74 (66.7)	57 (68.7)	17 (60.8)	*0.440*
ASA 4, N (%)	20 (18)	13 (15.7)	7 (25.0)	*0.260*
ASA 5, N (%)	4 (3.6)	2 (2.4)	2 (7.1)	*0.240*
Charlson Comorbidity Index (CCI) score				
Charlson 4, N (%)	25 (22.5)	20 (24.2)	5 (17.9)	*0.490*
Charlson 5, N (%)	37 (33.3)	28 (33.7)	9 (32.1)	*0.870*
Charlson 6, N (%)	28 (25.2)	22 (26.5)	6 (21.4)	*0.590*
Charlson 7, N (%)	10 (9.1)	5 (6)	5 (17.9)	*0.060*
Charlson 8, N (%)	7 (6.3)	5 (6)	2 (7.1)	*0.830*
Charlson 9, N (%)	1 (0.9)	1 (1.2)	0 (0.00)	*0.560*
Charlson 10, N (%)	2 (1.8)	1 (1.2)	1 (3.6)	*0.420*
Charlson 11, N (%)	1 (0.9)	1 (1.2)	0 (0.0)	*0.560*
Comorbidities				
Diabetes, N (%)	22 (19.8)	17 (20.5)	5 (17.9)	*0.740*
COPD, N (%)	14 (12.6)	9 (10.8)	5 (17.9)	*0.330*
Hypertension, N (%)	68 (61.3)	53 (63.8)	15 (53.6)	*0.330*
Chronic Renal Failure, N (%)	16 (14.4)	10 (12)	6 (21.4)	*0.220*
Ischemic Heart Disease, N (%)	26 (23.4)	22 (26.5)	4 (14.3)	*0.190*
Valvular Heart Disease, N (%)	10 (9)	4 (4.8)	6 (21.4)	***0.008***
Open Abdominal Surgery, N (%)	36 (32.4)	26 (31.3)	10 (35.7)	*0.670*
Laparoscopic Abdominal Surgery, N (%)	41 (36.9)	30 (36.1)	11 (39.3)	*0.770*
Provenance before admission				
Home, N (%)	61 (54.9)	51 (61.4)	10 (35.7)	***0.018***
Nursing Home, N (%)	12 (10.8)	8 (9.6)	2 (7.1)	*0.690*
Conventional Hospital Department, N (%)	24 (21.7)	16 (19.3)	8 (28.6)	*0.300*
ICU, N (%)	14 (12.6)	6 (7.2)	8 (28.6)	***0.003***
Vital parameters				
Heart Rate, Bpm	89.3 ± 18.8 [59–150]	89.4 ± 17.7 [59–150]	89 ± 22.2 [60–141]	*0.260*
Systolic Blood Pressure, Mmhg	122 ± 27.7 [60–193]	124.5 ± 27.7 [60–193]	116.2 ± 27.0 [66–179]	*0.620*
Diastolic Blood Pressure, Mmhg	70 ± 18 [29–126]	71.9 ± 16.7 [35–126]	64 ± 20.8 [29–116]	*0.250*
Oxygen Saturation Level, %	94.8 ± 4.9 [52–100]	95.2 ± 2.5 [86–100]	93.6 ± 9.1 [52–100]	*0.140*
Temperature, °C	36.98 ± 0.9 [33.8–39.2]	37 ± 0.8 [35–39.2]	36.7 ± 1.3 [33.8–38.6]	*0.064*
Sepsis Condition, N (%)	28 (25.2)	14 (16.9)	14 (50)	***0.001***

BMI, Body Mass Index; ASA, American Society of Anesthesiology; COPD, Chronic Obstructive Pulmonary Disease; ICU, Intensive Care Unit

Twenty-eight patients presented with sepsis upon admission (25.2%) while thirty-six patients presented with shock upon admission (32.4%), requiring immediate hospitalization in the intensive care unit before surgery. Table 2 shows detailed laboratory data.

**Table 2:** Biological values upon admission

Variable, Mean [range]	Overall population (n = 111)	Survivor (n = 83)	Non-Survivor (n = 28)	*p*
CRP (Mg/L)	130.3 ± 120.8 [4–512]	117.4 ± 115.7 [4–512]	176.6 ± 129.7 [7.7–426]	*0.460*
Leucocyte Count, g/l	13.3 ± 6.8[2.3–37]	13.2 ± 6.8 [2.3–37]	13.5 ± 6.8 [3.4–32.4]	*0.820*
Hemoglobin, g/dl	12.1 ± 2.6 [6.7–19.8]	12.2 ± 2.4 [6.7–17.1]	11.9 ± 2.9 [9–19.8]	*0.340*
Platelets Count, g/l	293 ± 120.4 [8.1–660]	298.6 ± 120 [107–660]	276.2 ± 122.2 [8.1–545]	*0.970*
Creatinine Level, µmol/l	122 ± 87.8 [35.2–466.9]	106.3 ± 72.5 [40.4–430]	171.9 ± 110.3 [35.2–466.9]	***0.006***
Bilirubin Level, µmol/l	15.4 ± 13.5 [2.3–96]	14.1 ± 7.8 [2.8–42.9]	19.3 ± 22.9 [2.3–96]	*0.277*
Lactate Level, µmol/l	3.3 ± 2.8 [0.8–13]	2.7 ± 1.8 [0.9 − 7.3]	4.9 ± 4 [0.8–13]	***0.010***

CRP, C Reactive Protein

Indications for surgery included: complicated sigmoiditis in 38 patients (34.3%), cancer in 35 patients (31.5%), ischemic colitis in 31 patients (27.9%), complicated appendicitis in 1 patient (0.9%), strangulated hernia in 2 patients (1.8%), bleeding in 4 patients (3.6%) (Table 3). Left colectomy was the most performed surgical procedure in nearly half of the population (53 patients, 47.7%), followed by right colectomy (26 patients, 23.4%), total colectomy (13 patients, 11.7%), and ileocecal resection (10 patients, 9%).

**Table 3:** Operative data

Variable	Overall population (n = 111)	Survivor (n = 83)	Non-Survivor (n = 28)	*p*
Diagnosis				
Complicated Sigmoiditis, N (%)	38 (34.3)	29 (34.9)	9 (32.2)	*0.970*
Cancer, N (%)	35 (31.5)	29 (34.9)	6 (21.4)	*0.180*
Ischemic Colitis, N (%)	31 (27.9)	18 (21.7)	13 (46.4)	***0.012***
Strangulated Hernia, N (%)	2 (1.8)	2 (2.4)	0 (0.0)	*0.410*
Appendicitis, N (%)	1 (0.9)	1 (1.3)	0 (0.0)	*0.560*
Digestive Hemorrhage, N (%)	4 (3.6)	4 (4.8)	0 (0.0)	*0.410*
Perioperative data				
Right Colectomy, N (%)	26 (23.4)	20 (24.1)	6 (21.4)	*0.770*
Ileo-Caecal Resection, N (%)	10 (9)	7 (8.4)	3 (10.7)	*0.710*
Transverse Colectomy, N (%)	2 (1.8)	2 (2.4)	0 (0.0)	*0.410*
Left Colectomy, N (%)	53 (47.7)	43 (51.8)	10 (35.7)	*0.140*
Sub-Total Colectomy, N (%)	5 (4.5)	2 (2.4)	3 (10.7)	***0.070***
Total Colectomy, N (%)	13 (11.7)	7 (8.4)	6 (21.4)	***0.060***
Associated Resection, N (%)	12 (10.8)	7 (8.4)	5 (17.9)	*0.170*
Restoration Of Continuity, N (%)	23 (20.7)	20 (24.1)	3 (10.7)	*0.130*
Ileostomy, N (%)	36 (32.4)	22 (26.5)	14 (50)	***0.022***
Colostomy, N (%)	51 (45.9)	41 (49.4)	10 (35.7)	*0.210*
Laparotomy, N (%)	101 (90.9)	74 (89.2)	27 (96.4)	*0.250*
Laparoscopy, N (%)	10 (9.1)	9 (10.8)	1 (3.6)	*0.250*
Temporary Laparostomy, N (%)	5 (4.5)	1 (1.2)	4 (14.3)	***0.004***
Malignant Pathology, N (%)	35 (31.5)	27 (32.5)	8 (28.6)	*0.700*
Operating Time, Mn	146 ± 59	132 ± 54	159 ± 7	*0.150*

In nearly 80% of cases, an ostomy was performed (87 patients, 78.3%), and particularly a colostomy in 51 patients (45.9%) and an ileostomy in 36 patients (32.4%) respectively, while immediate restoration of bowel continuity was performed in 23 patients (20.7%). A total of 101 procedures were performed via laparotomy (90.9%) while a laparoscopic approach was used in 10 patients (9.1%). The overall mean operating time was 146 ± 59 min [19–349]. Mean laparotomic time was 149 ± 59 min [19–349] while mean laparoscopic time was 120 ± 51 min [33–229] (p = 0.15). More detailed operative data are reported in Table 3.

### Postoperative results

After surgery, 62 patients (55.8%) were admitted to the intensive care unit with a mean length of hospital stay of 3.7 ± 5.8 days [1–29]. Mean total in-hospital length of stay was 17.92 ± 16.07 days [1-128].

Eighteen patients (16.2%) presented an uneventful postoperative course; 37 patients (33.3%) presented a minor complication (Clavien-Dindo I: 7 patients; Clavien-Dindo II: 30 patients), while 56 patients (50.4%) presented a major complication (Clavien-Dindo IIIa: 4 patients; Clavien-Dindo IIIb: 12 patients; Clavien-Dindo IV: 12 patients; Clavien-Dindo V: 28 patients). The overall 30-day mortality rate was 25.2%.

Notably, 51.6% of patients (48 cases) who presented at least one complication suffered from multiple complications.

Reintervention was necessary in 11 patients (9.9%). Four patients developed an ileocolonic anastomotic leakage requiring surgical revision and the confection of an ileocolostomy (on postoperative days 3, 5, 6, and 7, respectively). Three patients were reoperated on for postoperative evisceration (on postoperative days 5, 8, and 15, respectively), while in one case a persistent postoperative ileus on postoperative day 20 after a laparoscopic Hartmann procedure required an extensive adhesiolysis via laparotomy. Finally, one pelvic abscess following a Hartmann procedure required a revision for surgical lavage/drainage on postoperative day 12. Details on complications are reported in Table 4.

**Table 4:** Postoperative complications

Complication grade	N (%)	Complication grade	N (%)
**Dindo I**	**7 (6.3)**	**Dindo IIIa**	**4 (3.6)**
*Wound abscess*	3 (2.7)	*Intraabdominal collections necessitating percutaneous drainage*	3 (2.7)
*Acute renal failure*	2 (1.8)	*Arrythmia necessitating Pacemaker placement (POD 9)*	1 (0.9)
*Diarrhea*	1 (0.9)	**Dindo IIIb**	**12 (10.8)**
*Hypokalemia*	1 (0.9)	*Anastomotic leak (POD3, POD5, POD6 and POD7)*	4 (3.6)
		*Evisceration*	1 (0.9)
		*Perforated acute cholecystitis (POD6)*	1 (0.9)
**Dindo II**	**30 (27)**	*Persistent postoperative ileus requiring reintervention (POD20)*	1 (0.9)
*Postoperative ileus (requiring NGT placement)*	11 (9.9)	*Pelvic abscess caused by rectal stump leakage*	1 (0.9)
*Urinary infection*	1 (0.9)	*Hemorrhagic gastroduodenal ulcer necessitating endoscopic hemostasis*	2 (1.8)
*Splenic infarct*	1 (0.9)	*Colic ischemia necessitating SMA stenting*	1 (0.9)
*Cardiac decompensation*	5 (4.5)	*Angiocholitis treated by ERCP (POD8)*	1 (0.9)
*Rectal bleeding (managed with simple transfusion)*	3 (2.7)		
*AF*	3 (2.7)	**Dindo IV**	**12 (10.8)**
*PE*	1 (0.9)	*Septic shock*	6 (5.4)
*DVT*	1 (0.9)	*Hemorrhagic shock (colo-rectal anastomotic bleeding treated endoscopically)*	1 (0.9)
*Urinary retention*	1 (0.9)	*ARDS*	2 (1.8)
*EAS*	1 (0.9)	*PE*	1 (0.9)
*malnutrition*	1 (0.9)	*AVC*	1 (0.9)
*Anemia*	1 (0.9)	*Acute renal failure requiring hemodialysis*	1 (0.9)
		**Dindo V**	**28 (25.2)**

AF, Atrial Fibrillation; PE, Pulmonary Embolism; DVT, Deep Vein Thrombosis; EAS, edemato-ascitic syndrome; POD, Postoperative Day; SMA, Superior Mesenteric Artery; ERCP, Endoscopic Retrograde Cholangio-Pancreatography; ARDS, Acute Respiratory Distress Syndrome

**Fig. 1:**
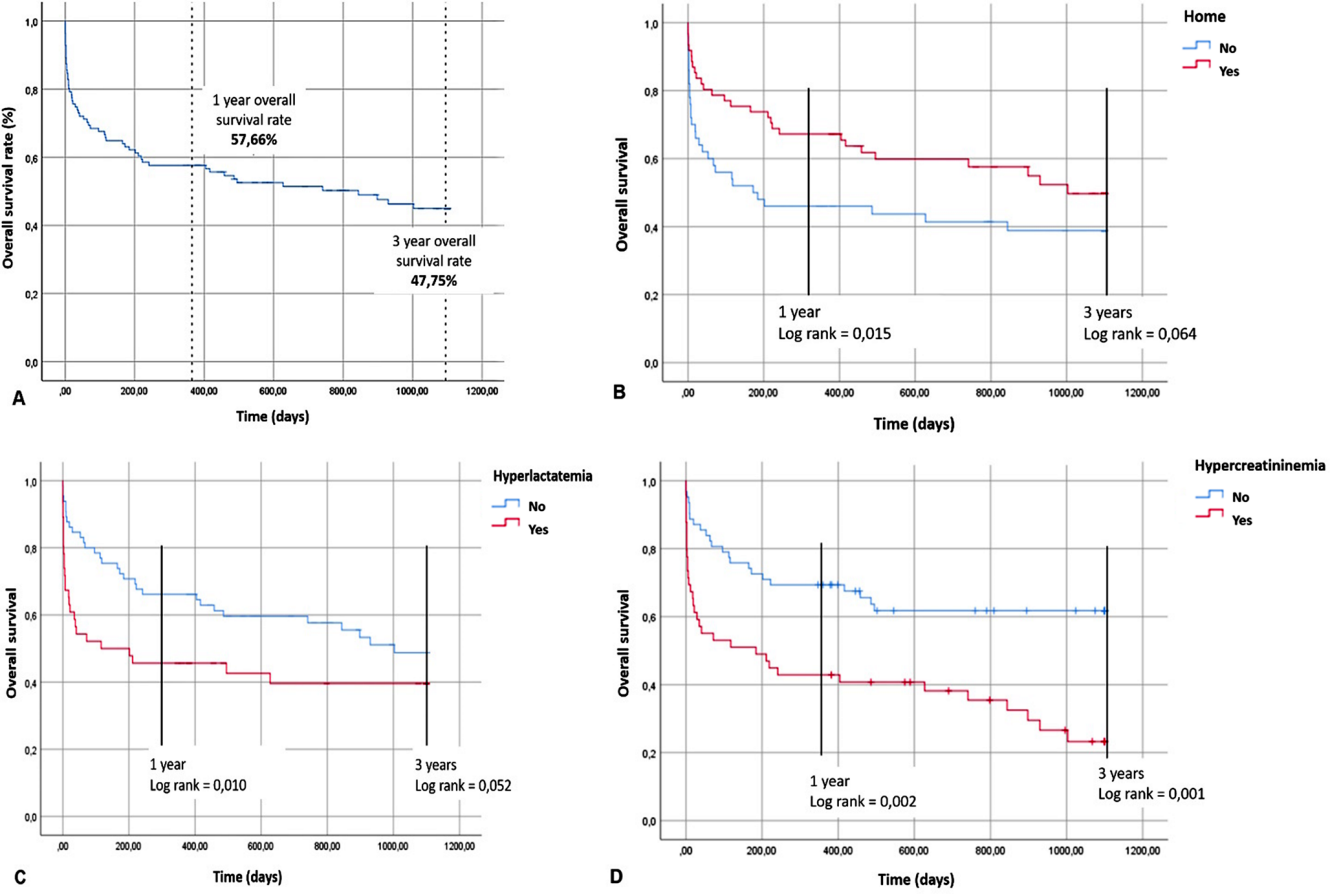
Overall survival (**A**) and impact of home origin (**B**), lactate level (**C**), and creatinine level (**D**) at 1- and 3-year survival

As abovementioned, the 30-day postoperative mortality was 25.2%, while the overall survival rate at 1 and 3 years was 57.6% and 47.7%, respectively. Mean follow-up was 23.6 ± 25 months (0.03-76) (Fig. 1A).

Univariate analysis of preoperative risk factors for mortality shows that the history of valvular heart disease (p = 0.008, OR: 5.39 [1.39–20.79]), intensive care unit provenance (p = 0.003, OR: 5.13 [1.60–16.50]), preoperative sepsis (p < 0.001, OR: 4.93 [1.93–12.50]), diagnosis of ischemic colitis (p = 0.012, OR: 3.13 [1.26–7.76]), creatinine (p = 0.006) and lactate levels (p = 0.01) were significantly associated with 30-day mortality, while patients coming from home had a lower 30-day mortality rate (p = 0.018, OR: 0.35 [0.14–0.85]).

Additionally, intraoperative variables associated with 30-day mortality included ileostomy creation (p = 0.022, OR: 2.78 [1.14–6.73]) and temporary laparostomy (p = 0.004, OR:13.67 [1.46–128.10]).

At multivariable analysis, only lactate (p = 0.032) and creatinine levels (p = 0.027) were found to be independent predictors of 30-day mortality, while provenance from home was found to be an independent protective factor (p = 0.004, OR: 0,1 [0.02–0.46]). Univariate and multivariate analysis results are summarized in Table 5.

**Table 5:** Uni- and Multivariate Analysis of factors predicting 30-days mortality

Variable	Survivor (n = 83)	Non-Survivor (n = 28)	Univariate analysis			Multivariate Analysis		
			*p*	*Odds Ratio*	*95% IC*	*p*	*Odds Ratio*	*95% IC*
Valvular Heart Disease, N (%)	4 (5)	6 (21)	***0.008***	5.39	1.39 − 20.79			
Provenance from home, N (%)	51 (61)	10 (35)	***0.018***	0.35	0.14 − 0.85	***0.004***	0.1	0.02–0.46
ICU provenance, N (%)	6 (7)	8 (28)	***0.003***	5.13	1.60 − 16.50			
Sepsis Condition, N (%)	14 (17)	14 (50)	***0.001***	4.93	1.93 − 12.50			
Creatinine Level, Mmol/L	106.32 [40.4–430]	171.86. [35.2–466.9]	***0.006***			***0.032***	1	1.001–1.01
Lactate Level, Mmol/L	2.7 [0.97 − 7.3]	4.96 [0.8–13.04]	***0.010***			***0.027***	1.31	1.001–1.72
ICU need on admission, N (%)	19 (22.89)	17 (60.71)	***0.001***					
Ischemic Colitis, N (%)	18 (22)	13 (46)	***0.012***	3.13	1.26 − 7.76			
Ileostomy, N (%)	22 (27)	14 (50)	***0.022***	2.78	1.14 − 6.73			
Temporary Laparostomy, N (%)	1 (1)	4 (14)	***0.004***	13.67	1.46–128.10			

ICU, Intensive Care Unit

Interestingly, the negative prognostic value of hyperlactatemia and hypercreatinemia, as well as the positive prognostic value of provenance from home on overall survival is maintained at 1 year of follow-up (p = 0.01, p = 0.002, and p = 0.015, respectively), while only hypercreatinemia maintained its impact on overall survival at 3 years (p = 0.001). On the contrary, hyperlactatemia was not associated with increased mortality in the long term (p = 0.064 and p = 0.052) (Fig. 1, B, C, D).

## Discussion and conclusion

The population of elderly people is progressively increasing yearly, faster than any other population segment, especially in Western countries [1].

As a result, surgeons will daily increasingly deal with such patients who require a more complex management than younger patients. It is due to the higher prevalence of age-related comorbidities and an intrinsic frailty related to a physiological decrease in basic functions of each organ with the aging process [9].

The emergency setting enhances such aspects, quickly unbalancing an already precarious equilibrium towards organ function impairment, thereby increasing the risk of intraoperative and postoperative complications.

For such reasons, an accurate preoperative risk assessment should be performed in these cases, in order to identify high-risk patients. Unfortunately, essential shared and detailed algorithms are not yet available.

Our analysis helped us to identify some variables associated with an increased risk of mortality.

Patients coming from a domestic environment appeared to present significantly lower 30-day mortality both in univariate and multivariate analyses, as well as regarding 1-year survival rates. These results are related to a likely better psychophysical status in elderly patients living at home compared to aged patients who are hospitalized [10].

Regarding patient comorbidities, chronic renal impairment and valvular heart disease rates were higher in the group of patients deceased at 30 days, even if no statistical differences in major comorbidities were found between the two groups. In this respect, similar results were found in the literature [11, 12]. However, the retrospective nature of our study, as well as the small sample size might well account for the lack of statistical significance, and further studies are needed to assess the real impact of comorbidities on short-term mortality.

The ASA score tends to be higher in patients who died within 30 days after surgery. This trend has already been confirmed in several studies [12, 13] such as the series by Lavanchy et al. [13], which showed that an ASA score ≥ 4 was a significant predictor of 30-day mortality with an odds ratio of 11.

No significant differences in vital parameters on admission between patients who died at 30 days and those who survived were evidenced in our series, consistently with the results of Lavanchy et al. [13]. However, direct admission to the intensive care unit was an independent risk factor for 30-day mortality. In our study, the amount of patients who needed an intensive care unit admission before surgery was significantly higher in the group of patients who died within 30 days (60.7%, 17 patients) as compared to the groups of 30-day survivors (22.9%, 19 patients) (p = 0.001). Additionally, half of the patients who died within 30 days presented a septic condition at admission, as compared to only 17% of the survivors (p = 0.001). However, these two factors did not maintain significance in multivariate analysis, probably due to the limited number of patients in our series.

In our analysis, hyperlactatemia and hypercreatinemia on admission were found to be independent risk factors for 30-day mortality, consistently with what was reported in the literature [13, 14]. Accordingly, we could have expected that the diagnosis of mesenteric ischemia would also have resulted in an independent risk factor. Indeed, the trend found in univariate analysis was not confirmed in multivariate analysis. Modini et al. [12] found similar results reporting that ischemic disease was significantly associated with 30-day mortality in univariate analysis but not in multivariate analysis in octogenarians. Conversely, Lavanchy et al. [13] showed that mesenteric ischemia was a predictive factor for 30-day mortality (p < 0.001, OR: 52.6 [8.93–309.94]). However, in their study, the authors included a more variegated spectrum of emergency surgery indications (e.g., cholecystitis, small bowel obstruction, etc.) than strictly colorectal cases.

Patients with malignancy are generally considered more fragile. In our analysis, colonic malignancy was not found to be a predictive factor of 30-day mortality and did not impact the 1- and 3-year survival. It is a major point since approximately 40% of patients with colonic cancer are aged over 75 [15], and studies have shown that octogenarians and nonagenarians account for a quarter of patients undergoing colorectal cancer surgery (scheduled or emergency) [2]. Consequently, emergency surgery for colorectal cancer in octogenarians should not be considered a contraindication [16]. In line with that, several authors failed to identify malignancy as a risk factor for early mortality, such as Sharrock et al. [17] Mamidanna et al. [18], and Modini et al. [12]. Notably, in these series, the 48, 47, and 43% of colorectal cancer patients were treated in an emergency setting. Our rate of malignancy (28.8%) is lower than other older series reported in the literature [12, 17, 18]. This could be partially due to the improvement and consolidation of colorectal cancer screening programs in recent years, allowing for an early management of colorectal cancer [19].

The overall 30-day complication rate was 83.8% (93 patients), almost in line with what was predicted by the POSSUM score analysis (79.3%). Comparable rates are reported in similar series of emergency colorectal resections [16, 20]. Some other series addressing emergency surgery in the elderly present lower morbidity rates, such as the one by Fukuda et al. (overall morbidity rate of 44%) [21]. However, in their study, the authors did not exclusively focus on colorectal surgery, including other emergency general surgery procedures, such as cholecystectomy or hernia repair that notably present lower complication rates.

The anastomotic leak rate was found to be 3.6% (4 patients). All patients required reintervention. This rate is slightly lower than other reports found in the literature, ranging from 4.7% in the series by Modini et al. [12] to 6.3% in the study by Iversen et al. [22].

Lehmann [23] reported the results of anastomotic leakage and death in elective surgery for left-sided colorectal cancer in elderly patients and did not find differences of leak rate (8.6 vs. 9.7%) between patients older or younger than 80 years (p = 0.084). However, age was an independent factor for not receiving the anastomosis and associated with death. In patients with leakage, the 2-year overall survival was significantly different between older and younger patients. Nevertheless, this study excluded emergency cases from their analysis.

In our study, the 30-day mortality rate was 25.2%, which is lower than the probability predicted with the POSSUM score (35.9%). This finding suggests the possibility that the POSSUM score might not be adapted for the population of elderly patients [24], and it might overestimate the mortality risk in emergency settings [25]. Globally, our findings are consistent with what was reported in the literature, with a mortality rate even lower than other authors’ experiences, such as Modini’s et al. [12], Iversen’s et al. [22], and Green’s et al. [20] who reported a 30-day mortality rates of 30, 35, and 44%, respectively. In a recent study [26] originating from a nationwide analysis, geriatric patients were found to have a higher mortality rate after elective colorectal cancer surgery not associated with the leak rate and having almost five times higher odds for deaths when compared to the baseline age group below 60 (OR: 4.86, 95% CI: 4.45–5.53, p < 0.001). However, in hospital mortality was significantly lower in patients operated on in high-volume centers (9.1%) versus 12.1% of patients operated on in low-volume centers (p < 0.001). This suggests that these parts of the population should be addressed in experienced centers. We had higher mortality rates in an emergency setting, and it was not associated with patient age at univariate and multivariate analyses.

Overall patient follow-up was 23.6 months, which is one of the longest follow-up ever reported for this subgroup of patients in the literature [12, 20, 27]. Globally, one-year and 3-year overall survival rates observed in our series of 57.6 and 47.7% of patients respectively highlight the rationale for the surgical management of these patients.

It is still a morbid surgery, and the surgical indications should be adequately evaluated. Additionally, preoperative global patient health status evaluation is critical, and various ethical considerations must be taken into account in emergency settings in the elderly patient [28].

However, it seems difficult to find predictive factors of early mortality in geriatric colorectal surgery. Hyperlactatemia and hypercreatinemia appear to be independent predictors of 30-day mortality. Admission from home appears to be a positive factor, probably because of a better general health condition. Our findings seem to corroborate the evidence reported by other series, identifying some preoperative criteria associated with mortality. Such results would need to be validated by further larger studies to ideally develop a sort of predictive score that can stratify patients according to the early postoperative mortality risk, thereby helping surgeons with a decisional algorithm.

Very interestingly, some factors influencing short-term mortality outcomes continue to predict mortality at one year (hypercreatinemia, hyperlactatemia, and home provenance, p = 0.01, p = 0.002, and p = 0.015, respectively) and at three years (hypercreatinemia, p = 0.001). This aspect should be considered and included in the decision-making process.

The strongest points of our study include the longest follow-up in the literature for this type of study and one of the lowest reported 30-day mortality rate. The limitations of this study lie in its retrospective and monocentric nature, as well as in the lack of possibility to develop a predictive score. However, a prospective clinical trial would be unrealistic considering the context of emergency surgery. As a conclusion, emergency colorectal surgery in octogenarians appears to be feasible without being unreasonable. Age should not be a contraindication for surgery. The presence of a malignant pathology should not be an obstacle to management.

## Data Availability

The data that support the findings of this study are available on request from the corresponding author.

## Declarations

### Permission to reproduce material

No material has been reproduced from other sources.
